# Gut mycobiome and neuropsychiatric disorders: insights and therapeutic potential

**DOI:** 10.3389/fncel.2024.1495224

**Published:** 2025-01-08

**Authors:** Ines Hadrich, Mariem Turki, Imen Chaari, Balkiss Abdelmoula, Rahma Gargouri, Nahed Khemakhem, Dhawia Elatoui, Fatma Abid, Sonda Kammoun, Mona Rekik, Samir Aloulou, Mariem Sehli, Aymen Ben Mrad, Sourour Neji, Fabian M. Feiguin, Jihene Aloulou, Nouha Bouayed Abdelmoula, Hayet Sellami

**Affiliations:** ^1^Fungal and Parasitic Molecular Biology Laboratory LR 05ES11, Faculty of Medicine, University of Sfax, Sfax, Tunisia; ^2^Psychiatry “B” Department, Hedi Chaker University Hospital, Sfax, Tunisia; ^3^Reserach Unit “Drosophila”UR22ES03, Faculty of Medicine, University of Sfax, Sfax, Tunisia; ^4^Genomics of Signalopathies at the Service of Precision Medicine LR23ES07 FMS, University of Sfax, Sfax, Tunisia; ^5^Department of Pneumology, Faculty of Medicine, University of Sfax, Sfax, Tunisia; ^6^Ophthalmology Department, Habib Bourguiba University Hospital, Faculty of Medicine, Sfax, Tunisia; ^7^Medical Carcinology Department, Mohamed Ben Sassi University Hospital of Gabes, Faculty of Medicine, Sfax, Tunisia; ^8^Department of Life and Environmental Sciences, University of Cagliari, Cagliari, Italy; ^9^Parasitology and Mycology Laboratory - Habib Bourguiba University Hospital, Sfax, Tunisia

**Keywords:** gut mycobiome, neuropsychiatric disorders, dysbiosis, gut-brain axis, fungi, innovative therapy

## Abstract

**Background:**

The human gut mycobiome, a minor but integral component of the gut microbiome, has emerged as a significant player in host homeostasis and disease development. While bacteria have traditionally been the focus of gut microbiome studies, recent evidence suggests that fungal communities (mycobiota) may also play a crucial role in modulating health, particularly in neuropsychiatric disorders.

**Objective:**

This review aims to provide a comprehensive overview of current knowledge on the relationship between the gut mycobiome and neuropsychiatric disorders, exploring the potential of targeting fungal communities as a novel therapeutic strategy.

**Methods:**

We summarized recent findings from metagenomic analyses that characterize the diversity and composition of gut mycobiota and discuss how these communities interact with the host and other microorganisms via the gut-brain axis. Key methodologies for studying mycobiota, such as high-throughout sequencing and bioinformatics approaches, were also reviewed to highlight advances in the field.

**Results:**

Emerging research links gut mycobiota dysbiosis to conditions such as schizophrenia, Alzheimer’s disease, autism spectrum disorders, bipolar disorder, and depression. Studies indicate that specific fungal populations, such as *Candida* and *Saccharomyces*, may influence neuroinflammation, gut permeability and immune responses, thereby affecting mental health outcomes.

**Conclusion:**

Understanding the gut mycobiome’s role in neuropsychiatric disorders opens new avenues for therapeutic interventions, including antifungal treatments, probiotics, and dietary modifications. Future research should integrate multi-omics approaches to unravel the complex interkingdom interactions within the gut ecosystem, paving the way for personalized medicine in mental health care.

## 1 Introduction

Over the past decade, the microbiota and its effect on health has been largely studied. Gut microbiota have been recognized as a crucial key regulator of health status, and, consequently, its alterations can lead to several diseases (D’Argenio et al., 2022). Researchers have primarily focused on gut bacterial microbiota, often overlooking the role of the fungal, the viruses and parasites fractions. In fact, despite the small fraction of fungi approximately 0.1% of the total microbial population in the gut (Chin et al., 2020), the secondary metabolites produced during interactions with bacteria and host cells are essential for immunological control and metabolic homeostasis.

A growing body of research highlighted the close relationship between gut microbiota and psychiatric disorders, with dysbiosis playing a pivotal role in conditions like depression, anxiety, Autism spectrum disorder (ASD), Attention-deficit/hyperactivity disorder (ADHD), and schizophrenia (SCZ) (Ahmed et al., 2024). In fact, dysbiosis of the normal gut microbiota can have negative consequences for humans, particularly during critical stages of life, as the gut microbes change with age in both phenotype and number of bacterial species. Alterations in the metabolites produced by the gut microbiota can impact neurological disorders, mental health conditions, and even euthymic states (Jones et al., 2019). Emerging evidence highlights that the gut mycobiome, like its bacterial counterpart, influences the microbiota-gut-brain axis and mental health outcomes, with shifts in fungal communities linked to conditions such as SCZ, mood disorders, and ASD.

In this review, we discuss the technological limitations in studying the gut mycobiota and summarized the recent findings on links between gut mycobiome and neuropsychiatric disorders.

## 2 Metagenomic mycobiota analysis

Analysis of fungal (mycobiota) diversity over the past decade has provided significant insights into the role of microbial communities in human diseases (Cui et al., 2013; Kumpitsch et al., 2019; Tiew et al., 2020). The results of microbial diversity studies are heavily influenced by the methods. Innovative technologies such as next-generation sequencing methods, and metagenomic and amplicon sequencing, have revolutionized our ability to identify and characterize fungal species in the human body.

However, data on fungal communities are still limited, partly because fungi are more challenging to manipulate and analyze (Angebault et al., 2020).

Metagenomic study of mycobiota in human fecal samples involves several steps, including sample collection, nucleic acid extraction, library preparation, sequencing and bioinformatics analysis. Samples must be handled in sterile environments, with rapid refrigeration at -80°C to preserve microbiota diversity (Choo et al., 2015; Chiappori et al., 2022; Wang L.-J. et al., 2023). Nucleic acid extraction must effectively capture all fungi, despite the challenge of breaking the tough fungal cell wall, often requiring mechanical or enzymatic disruption (Nguyen et al., 2015; Halwachs et al., 2017; Vesty et al., 2017). For fungal profiling, both internal transcribed spacer (ITS), ITS1 and ITS2 sequencing are commonly used. Some studies have compared ITS1 and ITS2 for fungal profiling (Bazzicalupo et al., 2013; Blaalid et al., 2013; Monard et al., 2013; Wang et al., 2015; Yang et al., 2018). While some researchers found ITS2 to be more suitable for revealing richness (Bazzicalupo et al., 2013), others reported that ITS1 was likely the better choice for studying fungal and eukaryotic species (Yang et al., 2018).

Functional classification of metagenomics data is vital for investigating the functional and metabolic roles of microbiome member species, as well as their variations under different conditions/treatments. Overall, tools for functional classification share common features with tools used for whole genome analyses. Bioinformatics tools are essential for analyzing metagenomic data, including pre-processing, taxonomic classification, and statistical analysis. Various approaches, such as OTU clustering, can affect the results, and improving fungal databases is key to enhancing data accuracy. Tools like QIIME and mothur are commonly used, with specialized pipelines like LotuS and FindFungi improving fungal sequence classification (Donovan et al., 2018). Newer pipelines like DAnIEL support ITS analysis and allow for comparison with publicly available datasets (Loos et al., 2021).

These advancements in methodology and technology are crucial for studying the mycobiota and its potential links to brain health and behavior.

## 3 Mycobiome and neuropsychiatric disorders

A summary of the key studies on the gut mycobiome’s role in neuropsychiatric disorders, including their methodologies and main findings, is presented in Table 1.

**TABLE 1:** Summary of key studies on the gut mycobiome and neuropsychiatric disorders.

References	Methodology	Main findings
Yuan et al., 2024	Internal transcribed spacer (ITS) sequencing of gut samples	Found significant alterations in intestinal mycobiota composition in schizophrenia patients, suggesting dysbiosis may contribute to inflammation.
Yuan et al., 2023	18S rRNA sequencing and polygenic risk score (PRS) analysis	Higher Fungal Microbial Dysbiosis (F_MD) index and lower α-diversity were associated with increased schizophrenia risk; *Aspergillus* abundance positively correlated with cognitive functions.
Yuan et al., 2022	18S rRNA gene amplicon sequencing	Schizophrenia patients showed reduced fungal α-diversity and altered fungi-to-bacteria ratio; *Purpureocillium* abundance linked to severe psychiatric symptoms.
D’Argenio et al., 2022	Gut mycobiome analysis in 3xTg-AD mice model	Increased *Dipodascaceae* family potentially contributes to Alzheimer’s disease through metabolic alterations.
Nagpal et al., 2020	Fungal rRNA ITS1 gene sequencing before and after dietary intervention	Modified Mediterranean ketogenic diet (MMKD) reduced abundance of harmful fungi and increased beneficial fungi in mild cognitive impairment (MCI) patients.
Phuna and Madhavan, 2022	Analysis of brain fungal species in Alzheimer’s patients	Found common fungal species (e.g., *Candida* spp., *Malassezia* spp.) associated with neuroinflammation and neurodegeneration.
Jiang et al., 2020	Metagenomic and metabolomic analysis in depressive patients	Altered gut mycobiota composition and disrupted bacteria-fungi interactions associated with depressive episodes.
McGuinness et al., 2024	Systematic review of gut mycobiome studies in depression and bipolar disorder	Imbalances in fungal populations (e.g., increased *Candida* levels) linked to neuroinflammation and mood dysregulation, suggesting therapeutic potential for targeted microbiome interventions.
Wang L.-J. et al., 2023	Next-generation sequencing (NGS) platforms in ADHD patients	Found significant differences in fungal community composition; higher *Candida* abundance linked to increased intestinal permeability in ADHD patients.
Strati et al., 2017	Comparative mycobiome analysis in ASD and neurotypical controls	ASD samples exhibited reduced fungal diversity; predominance of *Debaryomycetaceae* and absence of certain fungal families compared to controls, indicating potential for targeted therapies.
Alharthi et al., 2022	Review on microbiota and ASD	Emphasized the interplay between gut bacteria and fungi, highlighting a potential microbial imbalance contributing to ASD pathology.
Wang C. et al., 2023	Animal model study on alcohol dependence and gut dysbiosis	Alcohol-dependent patients’ gut microbiome exacerbated anxiety- and depression-like behavior in rats, suggesting fungal dysbiosis impacts addiction behaviors.
Chu et al., 2019	Fungal exotoxin study in alcoholic hepatitis models	*Candida albicans* exotoxin *Candidalysin* damaged hepatocytes and increased liver inflammation, worsening alcoholic liver disease in mouse models.

### 3.1 Schizophrenia

Schizophrenia (SCZ) is a chronic and severe mental disorder characterized by relapsing episodes, prolonged course, and residual symptoms. Symptoms of SCZ fall into three main categories: psychotic, negative, and cognitive. Psychotic symptoms include hallucinations—sensory experiences such as seeing, hearing, or feeling things that are not present—and delusions, involving strong, often irrational beliefs. Negative symptoms encompass reduced motivation, diminished interest in daily activities, social withdrawal, and challenges in emotional expression and daily functioning. Cognitive symptoms typically include difficulties with attention, concentration, and memory.

Prior studies have increasingly focused on the microbiome–gut–brain axis, a complex communication system linking the gastrointestinal (GI) tract to the brain, in relation to SCZ (Ju et al., 2023). While considerable attention has been directed at the bacterial components of the gut microbiota in SCZ, the fungal aspect, or mycobiome, remains less explored. Nonetheless, emerging evidence suggests a potential link between gut mycobiome dysbiosis and SCZ development.

Yuan et al. (2024) conducted an analysis of gut mycobiota in 109 chronic SCZ patients and 77 controls, identifying significant reductions in fungal diversity and an overrepresentation of *Candida* species linked to inflammatory processes. This study used ITS sequencing to profile fungal diversity. They observed significant alterations in the mycobiota composition in SCZ patients, marked by a reduced diversity of fungal species compared to healthy controls. This dysbiosis was characterized by a depletion of fungi, such as *Saccharomyces cerevisiae*, known for its immune-modulatory effects, potentially impacting gut homeostasis and immune regulation in these patients. On the other hand, *Candida albicans*, an opportunistic fungal species commonly found in the human GI tract, plays a central role in this fungal dysbiosis. The overgrowth of *Candida albicans* has been linked to chronic inflammatory states and increased gut permeability, promoting neuroinflammatory responses through the production of pro-inflammatory molecules such as β-glucans (Huffnagle and Noverr, 2013; Huang et al., 2024; Kearns, 2024; Villavicencio-Tejo et al., 2023; Yuan et al., 2024). Yuan et al. (2024) demonstrated that a *Candida*-dominant enterotype correlates with more severe psychotic symptoms and heightened systemic inflammation. In another study, Yuan et al. (2023) investigated the combined effects of genetic risk and mycobiome dysbiosis on SCZ risk among 137 drug-naïve, first-episode SCZ patients and 76 healthy controls. Patients with a higher Polygenic Risk Score (PRS), a measure quantifying the cumulative genetic predisposition to SCZ, exhibited a *Candida*-dominant enterotype (Yuan et al., 2023). These findings highlight the interplay between genetic predispositions, fungal dysbiosis, and disease severity. For a summary of observed dysbiosis patterns and their clinical implications, see Table 2.

**TABLE 2:** Summary of key findings on mycobiome alterations in neuropsychiatric disorders.

Disorder	Key fungal species	Observed dysbiosis patterns	Clinical implications
Schizophrenia	*Candida*	Overrepresentation of *Candida*, depletion of beneficial fungi	Linked to immune dysregulation and chronic inflammation, potentially exacerbating psychotic symptoms.
Mood Disorders (Bipolar disorder, Depression)	*Candida*	Increased *Candida* levels, reduced diversity of beneficial fungi	Neuroinflammatory processes, disruption of neurotransmitter pathways, and potential impacts on psychopharmacological treatment efficacy.
Autism Spectrum Disorders	*Saccharomycetaceae, Debaryomycetaceae*	Altered levels of Saccharomycetaceae and Debaryomycetaceae	Gastro-intestinal symptoms, potential neurobehavioral effects, and emerging potential for fungal biomarkers in diagnosis or symptom monitoring.
Attention-Deficit/Hyperactivity Disorder (ADHD)	*Candida albicans*	Higher abundance of *Candida albicans*	May affect gut permeability and influence neurodevelopmental pathways; potential for therapeutic interventions like probiotics.
Alzheimer’s disease	*Dipodascaceae, Botrytis*	Increase in Dipodascaceae, altered fungal profiles with Botrytis	Possible role in inflammation and metabolic dysregulation in AD; fungal biomarkers like Dipodascaceae could aid in patient stratification and therapeutic monitoring.
Alcohol dependence	*Candida, Pichia*	Dominance of *Candida* and *Pichia* species	Increased gut permeability, liver inflammation, impact on addiction behaviors.

Further research by Yuan et al. (2022) explored changes in mycobiome composition and fungi-bacteria interaction networks in 205 SCZ patients compared to 125 healthy controls, using 18S ribosomal RNA gene sequencing. The study found reduced fungal α-diversity in SCZ patients, along with an altered fungi-to-bacteria diversity ratio. Notably, higher levels of *Purpureocillium* were associated with more severe psychiatric symptoms and poorer cognitive function in SCZ patients, emphasizing the potential impact of specific fungal species on disease progression (Yuan et al., 2022).

In an earlier study, Zhang et al. (2020) examined gut mycobiota dysbiosis in drug-naïve SCZ patients using ITS1-based DNA sequencing, comparing 10 SCZ patients to 16 healthy controls. The results revealed reduced fungal alpha diversity and altered composition, with higher levels of *Chaetomium* and lower levels of *Trichoderma* in SCZ patients. Additionally, SCZ patients exhibited an intensified bacteria-fungi interaction network, suggesting disrupted interkingdom interactions within the gut microbiome, which may play a role in SCZ pathophysiology (Zhang et al., 2020).

Given the observed overrepresentation of *Candida albicans* and its role in systemic inflammation, therapeutic strategies such as antifungal agents or probiotics targeting fungal dysbiosis could help restore gut homeostasis. Early evidence suggests that probiotics like *Saccharomyces boulardii* may reduce fungal overgrowth and improve gut barrier integrity, potentially mitigating neuroinflammatory pathways linked to SCZ (Kelesidis and Pothoulakis, 2012). These findings underscore the potential of therapeutic strategies targeting fungal dysbiosis, such as probiotics, to mitigate inflammation and improve gut-brain axis health in SCZ.

A detailed overview of SCZ-related findings, methodologies, and main fungal communities identified is provided in Tables 1, 2.

### 3.2 Alzheimer’s disease

Alzheimer’s disease (AD) is a chronic neurodegenerative condition characterized by amyloid beta plaques, neurofibrillary tangles, and both central and systemic inflammation, primarily affecting the medial temporal lobe and associative neocortical regions. Recently, the gut microbiome has gained attention as a modifiable risk factor for AD due to its bidirectional communication with the brain. Dysbiosis of the gut microbiota has been associated with the initiation and progression of neuroinflammatory processes that underlie AD (Seo and Holtzman, 2024). Emerging research suggests that the fungal component of the gut microbiome, or mycobiome, may also play a significant role in AD pathology (Seo and Holtzman, 2024).

A study by D’Argenio et al. (2022) analyzed the mycobiome of 3xTg-AD mice, a widely used model for AD, revealing a notable increase in fungi from the *Dipodascaceae* family compared to wild-type mice. To analyze the fungal composition, D’Argenio et al. (2022) utilized ITS sequencing in their assessment of the mycobiome within AD mouse models, revealing significant fungal shifts related to AD pathology. This family has been linked to metabolic disruptions, as it is also elevated in obese individuals and correlates with adiposity and cholesterol levels (Mar Rodríguez et al., 2015). Given the metabolic dysregulation observed in AD, D’Argenio et al. (2022) hypothesized that *Dipodascaceae* overgrowth may contribute to AD’s metabolic profile. However, further studies are needed to confirm these findings and elucidate the specific mechanisms involved (D’Argenio et al., 2022).

Dietary adjustments have shown potential in modulating the gut microbiome, which could impact AD progression. Nagpal et al. (2020) conducted a study on older adults with mild cognitive impairment (MCI) who followed either a modified Mediterranean ketogenic diet (MMKD) or the American Heart Association Diet (AHAD) for 6 weeks. Mycobiome analysis through fungal rRNA ITS1 gene sequencing revealed that MCI patients had higher proportions of fungal families and genera, including *Sclerotiniaceae*, *Phaffomycetaceae*, and *Botrytis*, compared to cognitively normal (CN) individuals. Notably, the MMKD intervention had a more substantial impact on the mycobiome than the AHAD, particularly in reducing levels of *Botrytis*, which was elevated in MCI patients at baseline. Additionally, the MMKD intervention led to an increase in *Geotrichum* abundance and a reduction in *Saccharomyces*, *Zygosaccharomyces*, and *Aureobasidium*, changes that may contribute to an improved gut environment. This suggests that diet-based interventions could modulate the gut mycobiome in ways that may benefit cognitive health. The study also highlighted correlations between specific fungal genera and Alzheimer’s biomarkers in cerebrospinal fluid (CSF). For example, *Aspergillus* and *Meyerozyma* were negatively correlated with amyloid-beta 40 and positively with tau protein levels in both CN and MCI patients, while *Wallemia* showed a negative correlation with tau markers in MCI but not CN subjects. These fungal biomarkers could help stratify patients based on their mycobiome profiles, enabling personalized dietary or pharmacological interventions (Nagpal et al., 2020).

While biomarkers derived from the gut mycobiome offer insights into systemic changes, the detection of fungal species directly within AD brains highlights a more localized impact, further implicating fungal dysbiosis in AD pathology. Phuna and Madhavan detected *Candida spp., Malassezia spp., Cladosporium spp.*, and *Alternaria spp.* in AD brains, suggesting that these fungi may inadvertently reach the brain, especially in immunocompromised individuals, through a compromised epithelial barrier (Phuna and Madhavan, 2022). *Candida* species, for instance, may induce fungal glial granulomas with accumulated amyloid precursor protein, while *Malassezia* species can activate neuroinflammatory Th1 and Th17 immune responses. The roles of *Cladosporium* and *Alternaria* in AD are less understood, but these fungi may contribute to neuroinflammation and could potentially be involved in acetylcholinesterase inhibition. The presence of these fungi may collectively drive chronic neuroinflammation and neurodegeneration in AD. Additionally, targeting specific fungal biomarkers could provide a novel approach for early diagnosis and monitoring of disease progression (Nagpal et al., 2020).

Overall, these studies underscore the complex relationship between the gut mycobiome and AD, highlighting potential avenues for therapeutic interventions. Diet-based strategies, particularly those resembling the Mediterranean ketogenic diet, may offer benefits by modulating mycobiome composition. Additionally, mycobiome biomarkers could serve as tools for monitoring AD progression and treatment efficacy. As research progresses, a deeper understanding of these fungal contributions could lead to targeted approaches that address both bacterial and fungal dysbiosis in AD.

Key findings on fungal dysbiosis in AD, including methodologies and clinical implications, are summarized in Tables 1, 2.

### 3.3 Autism spectrum disorders

Autism Spectrum Disorders (ASD) are complex neurodevelopmental conditions characterized by repetitive behaviors, restricted interests, and significant challenges in communication and social interaction (CDC, 2024). Despite extensive research, the exact causes of ASD remain elusive, though it is widely accepted that both genetic and environmental factors contribute to its development.

Recent studies have underscored a strong link between gastrointestinal (GI) issues and the severity of ASD symptoms. Individuals with ASD frequently experience a higher prevalence of GI disturbances, prompting researchers to explore the gut-brain axis and its potential role in ASD pathophysiology (Adams et al., 2011; Wang et al., 2011). Specifically, the gut microbiota has become a focal point of interest. Emerging evidence suggests that gut dysbiosis may correlate with the severity of ASD symptoms. This dysbiosis often manifests as reduced bacterial diversity and a decrease in beneficial commensal bacteria (Hughes et al., 2018). The mycobiome, the fungal component of the microbiome, has also gained attention, despite being previously overlooked in gut-brain axis research (Iovene et al., 2017; Strati et al., 2017).

In ASD, the fungal families *Saccharomycetaceae* and *Debaryomycetaceae* have been observed at altered levels. *Saccharomycetaceae*, which includes common yeast species, may play a role in GI issues frequently reported in ASD, potentially through interactions that influence gut motility and permeability. The presence of *Debaryomycetaceae* may contribute to neurobehavioral symptoms by affecting microbial metabolites that impact neurodevelopmental pathways (Strati et al., 2017; Zou et al., 2021; Alharthi et al., 2022)?. These alterations suggest that fungal communities may play a role in the severity of ASD-related GI issues and behavioral symptoms. This study used next-generation sequencing (NGS) techniques to evaluate fungal diversity, identifying associations between specific fungal taxa, like *Saccharomycetaceae*, and autism symptom severity (Strati et al., 2017).

The role of *Candida* species in ASD appears limited compared to other taxa. In ASD, although no significant differences in overall *Candida* abundance were observed compared to typically developing children, *Candida albicans* exacerbated GI symptoms in some cases (Strati et al., 2017). Therefore, targeting the mycobiome could represent a promising therapeutic approach for alleviating GI symptoms in children with ASD, potentially reducing symptom severity and enhancing quality of life (Strati et al., 2017). For ASD, targeted therapies such as probiotics or carefully monitored antifungal agents could help alleviate symptoms. However, their application requires caution due to potential toxicity and antifungal resistance (Alookaran et al., 2022). Emerging research suggests that restoring fungal balance through diet or fecal microbiota transplantation (FMT) may also hold promise in improving ASD outcomes (Strati et al., 2017; Wang L.-J. et al., 2023). Developing improved research methodologies will be crucial to optimizing these treatments and ensuring their efficacy and safety in managing ASD symptoms (Lewandowska-Pietruszka et al., 2023). A summary of ASD-associated fungal species and their potential neurobehavioral impacts can be found in Table 2.

In a broader point of view, recent research has highlighted the complex interactions between the gut microbiota and mycobiota, suggesting that these interactions might contribute significantly to ASD symptom severity. For instance, imbalances between bacterial and fungal populations in the gut may affect host microRNA expression, which in turn could regulate bacterial growth and contribute to ASD development (Yap et al., 2021). Additionally, small non-coding RNAs (sncRNAs), including microRNAs (miRNAs) and piwi-interacting RNAs (piRNAs), have been detected in fecal samples from individuals with ASD, suggesting a potential mechanism by which gut dysbiosis could exacerbate ASD symptoms through transcriptional regulation and inflammatory processes (Chiappori et al., 2022). These findings suggest that targeting gut dysbiosis may indirectly modulate RNA-mediated inflammatory pathways, offering a novel avenue for therapeutic research.

### 3.4 Mood disorders: bipolar disorder and depression

#### 3.4.1 Mood disorders and fungal interactions

Dysbiosis within the gut mycobiome has been associated with neuroinflammation and neurotransmitter disruptions (specifically disruptions in serotonin and dopamine pathways), which are relevant to the pathophysiology of mood disorders such as depression and bipolar disorder (McGuinness et al., 2024). For instance, increased levels of *Candida* and reduced diversity of other beneficial fungi have been observed in mood disorder patients, which may exacerbate mood dysregulation. Therapeutic strategies that target these fungal imbalances, such as probiotics or antifungal agents, could help restore a healthier microbiome balance and offer new avenues for managing mood disorder symptoms (McGuinness et al., 2024). These interventions may work by reducing systemic inflammation and restoring neurotransmitter homeostasis, thus alleviating mood disorder symptoms.

Key findings and fungal species associated with mood disorders are listed in Table 1 and summarized by clinical implications in Table 2.

#### 3.4.2 Bipolar disorder

Bipolar disorder (BD), previously known as manic-depressive illness, is a mental health condition characterized by extreme mood swings, changes in energy levels, activity, and concentration, significantly impacting daily functioning. Emerging research on the gut-brain axis suggests a potential role of the gut mycobiome in the development of BD.

While most research on BD and the gut-brain axis has concentrated on bacterial communities, this section emphasizes the emerging role of fungal dysbiosis, particularly involving *Candida albicans*, in mood stabilization and neuroinflammation. *Candida albicans*, discussed as a key species in fungal dysbiosis, contributes to chronic inflammation and mood dysregulation in BD. This specific dysbiosis, characterized by reduced fungal diversity, highlights *Candida albicans* as a key species potentially contributing to chronic inflammation and mood dysregulation in affected patients (McGuinness et al., 2022). Current literature suggests a possible relationship between mycobiome composition changes and BD symptoms. Additionally, as the gut mycobiome may influence the absorption of psychopharmacological treatments, adjusting its composition could improve BD symptoms (Lai et al., 2022). Emerging evidence also indicates that mycobiome-based biomarkers could be valuable for diagnosing BD and predicting treatment responses. From a therapeutic perspective, interventions targeting the mycobiome, such as probiotics and FMT, have shown potential for stabilizing mood and reducing BD symptoms. Modulating the mycobiome presents a promising strategy for managing BD (McGuinness et al., 2022). Additionally, studies have explored the role of *Candida albicans* in cognitive deficits and inflammation, which are common in both BD and SCZ. These findings suggest that fungal dysbiosis contributes to neuroinflammation and neurotransmitter disruptions, potentially aggravating BD symptoms (Musumeci et al., 2022). Also, mycobiome-specific probiotics could complement psychopharmacological treatments by addressing gut inflammation and improving drug absorption (Nagpal et al., 2019). Finally, causal analyses have identified a genetic-level link between gut microbiota and BD risk, emphasizing the need to explore fungal contributions within this framework (Xu et al., 2023).

#### 3.4.3 Depressive episodes

Dysbiosis has been described in patients experiencing current depressive episodes (CDE). Metagenomic and metabolomic analyses have been employed to better understand the structure and potential functions of the gut mycobiome and its impact on CDE development. In patients with CDE, the gut mycobiota is marked by a relative reduction in alpha diversity and altered composition, particularly higher levels of *Candida* and lower levels of *Penicillium* compared to healthy controls. The gut microbiota in these patients also shows significant disruptions in the bacteria-fungi correlation network, indicating altered interkingdom interactions (Jiang et al., 2020). Additionally, the potential of microbial alterations as biomarkers for diagnosing depressive episodes and predicting treatment responses has been highlighted (Jiang et al., 2020). Further research has shown that the intricate interactions between bacteria and fungi in the gut are crucial for maintaining overall gut health, and disruptions in these interactions are common in depressive disorders. These disturbances can exacerbate depressive symptoms and contribute to the systemic inflammation often observed in depression (Jiang et al., 2020).

However, the bidirectional nature of these alterations raises important questions. It remains unclear whether fungal dysbiosis actively drives depressive pathophysiology or arises as a secondary effect of depression-related parameters such as altered diet, medication, and stress. Additionally, while *Candida* has been implicated in neuroinflammatory processes, the functional roles of other fungi, such as *Penicillium*, in gut-brain axis regulation remain speculative and warrant further investigation.

### 3.5 Other psychiatric disorders

#### 3.5.1 Attention-Deficit/Hyperactivity Disorder (ADHD)

Attention-Deficit/Hyperactivity Disorder (ADHD) is a common childhood mental disorder with still poorly understood pathophysiological mechanisms. ADHD is characterized by persistent patterns of inattention and/or hyperactivity-impulsivity that interfere with daily functioning and development. Recent studies suggest that gut mycobiome and immune dysfunction may influence brain functions and social behaviors associated with ADHD.

Research exploring the gut mycobiome in ADHD using next-generation sequencing (NGS) platforms has highlighted potential links between fungal dysbiosis and ADHD susceptibility. At the *phylum* level, ADHD patients showed a significantly higher abundance of *Ascomycota* and a lower abundance of *Basidiomycota* compared to controls. At the *genus* level, *Candida*, particularly *Candida albicans*, was notably more abundant in ADHD patients (Wang L.-J. et al., 2023). Higher levels of *Candida albicans* have been associated with increased intestinal permeability, potentially influencing neurodevelopmental processes through systemic inflammation (Wang L.-J. et al., 2023). Such dysbiosis in the fungal mycobiome may contribute to ADHD-related behavioral symptoms through gut-brain axis interactions. Consequently, interventions targeting fungal dysbiosis could prove beneficial. Probiotics or synbiotics tailored to reduce fungal overgrowth and enhance gut barrier function warrant further investigation (Kelesidis and Pothoulakis, 2012).

Overall, this fungal imbalance may influence neurodevelopmental pathways relevant to ADHD. This underscores the potential of gut-targeted therapies, such as probiotics or antifungal agents, to address gut permeability issues and support neurodevelopmental health in ADHD patients.

Details on fungal dysbiosis in ADHD are consolidated in Tables 1, 2.

#### 3.5.2 Alcohol dependence

The fungal dysbiosis associated with alcohol dependence is still under investigation, but it is hypothesized that fungi can exploit disrupted bacterial homeostasis, often leading to fungal overgrowth following bacterial dysbiosis (Kakiyama et al., 2014; Bajaj et al., 2017; Yan et al., 2017; Chu et al., 2019; Lang et al., 2020). In alcohol dependence, fungal genera such as *Candida* and *Pichia* are frequently overrepresented. *Candida*, particularly *Candida albicans*, is known to disrupt gut barrier function, leading to increased gut permeability and systemic inflammation that may exacerbate alcohol cravings. *Pichia* species are thought to thrive in the altered gut environments found in alcohol-dependent individuals, further disturbing microbial homeostasis and potentially impacting addiction pathways via inflammatory and neurochemical changes (Rosenbach et al., 2010; Wang et al., 2014; Yang et al., 2017). Recent experiments suggest that gut dysbiosis in alcohol-dependent patients can promote anxiety-like and depression-like behaviors in animal models, potentially facilitating alcohol dependence by influencing cholecystokinin (CCK) and related receptors (Wang C. et al., 2023). Altered gut microbiome composition in alcoholism, along with compromised intestinal barrier integrity, may also worsen central nervous system symptoms, leading to depression, anxiety, and alcohol cravings (Leclercq et al., 2014). Additionally, the role of fungal dysbiosis in alcoholic liver disease (ALD) has gained attention. Studies have found that *Candida* and *Pichia* are dominant in the mycobiomes of patients with ALD, with these fungi accumulating β-glucan in the liver, potentially driving inflammation and exacerbating liver disease (Yang et al., 2017). Furthermore, *Candida albicans* exotoxin, *Candida* lysin, has been shown to damage liver cells and increase inflammation in alcoholic mouse models, worsening liver disease (Chu et al., 2019). FMT or antifungal therapies targeting these fungal species could complement current addiction treatments by addressing gut-derived inflammation (Bibbò et al., 2020; Chu et al., 2020).

These findings highlight the potential of gut mycobiome modulation as an adjunct to existing treatments, offering a comprehensive approach to managing both the physiological and behavioral aspects of alcohol dependence. For a summary of mycobiome findings in alcohol dependence, including clinical implications of fungal dysbiosis, refer to Tables 1, 2.

## 4 Discussion

**FIGURE 1:**
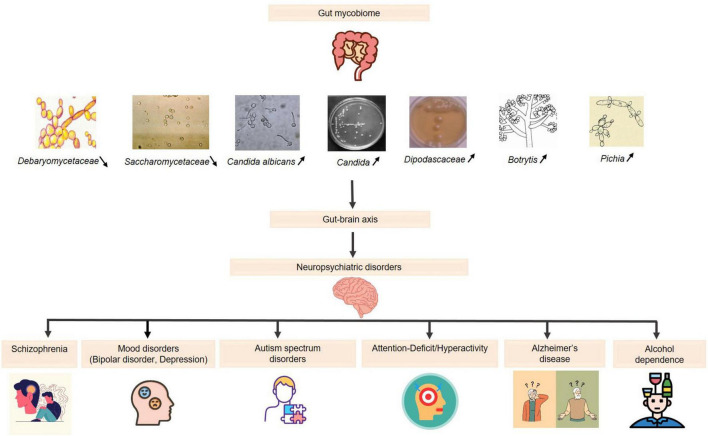
The role of the gut mycobiome in neuropsychiatric disorders via the gut-brain axis. Increased levels 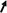: (*Candida*, *Candida albicans, Dipodascaceae, Botrytis, Pichia*): reduction in the diversity of beneficial fungi: immune system imbalance and disruption of neurotransmitter pathways → effectiveness of psychopharmacological treatments. Decreased levels 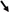: (*Saccharomycetaceae*, *Debaryomycetaceae*): affecting gut homeostasis → potential neurobehavioral consequences.

The gut mycobiome is emerging as a critical component of the gut-brain axis, with mounting evidence linking fungal dysbiosis to neuropsychiatric disorders such as SCZ, depression, and BD, as depicted in Figure 1. While bacterial microbiota has been extensively studied, fungi exhibit distinct biological properties, including the ability to persist in hostile environments, produce pro-inflammatory molecules like β-glucans, and interact directly with host epithelial and immune cells (Brown et al., 2003; Romani, 2011). These unique fungal traits may explain their role in modulating chronic inflammation and neurotransmitter pathways, differentiating their contributions from those of bacterial communities.

### 4.1 Key findings and remaining gaps

Current studies reveal a connection between reduced fungal diversity and mood-related disorders, suggesting that specific fungal taxa may influence disease progression. However, the causal relationships between fungal dysbiosis and neuropsychiatric outcomes remain unclear. Additionally, the interplay between fungal and bacterial communities in shaping the gut-brain axis is underexplored, limiting our ability to fully understand the synergistic effects.

While biomarkers derived from the gut mycobiome show promise for early diagnosis and treatment monitoring, their clinical utility remains unproven. Longitudinal studies and multi-omic approaches are urgently needed to validate these findings and establish the mechanistic pathways underlying these associations.

### 4.2 Therapeutic and clinical implications

Therapeutic strategies addressing fungal dysbiosis—such as probiotics, dietary interventions, or antifungal therapies—show promise in mitigating the impact of the gut mycobiome on neuropsychiatric disorders. These approaches, discussed in the specific sections, underscore the need for further clinical validation and personalized interventions. Incorporating the mycobiome as a biomarker, as highlighted in public health studies (Buytaers et al., 2024), could also pave the way for stratified therapies and better health monitoring in neuropsychiatric populations.

### 4.3 Future research directions

Advancing our understanding of the gut mycobiome in neuropsychiatric disorders will require comprehensive, interdisciplinary research. Multi-omic frameworks combining metagenomics, metabolomics, and transcriptomics should be prioritized to capture the complexity of fungal-bacterial interactions. Moreover, linking genetic predispositions, such as polygenic risk scores (PRS), with mycobiome data could enhance our understanding of disease susceptibility and progression. Finally, identifying reliable fungal biomarkers remains a critical objective for advancing diagnostic and therapeutic innovations in neuropsychiatry.

## 5 Conclusion

The gut mycobiome is an underexplored but critical aspect of the gut-brain axis, offering unique insights into the pathophysiology of neuropsychiatric disorders such as SCZ, depression, and BD. Recent studies have illuminated the potential role of fungal dysbiosis, particularly involving *Candida albicans*, in driving neuroinflammatory pathways, neurotransmitter imbalances, and mood dysregulation. These findings underscore the importance of integrating fungal communities into the broader microbiome research framework, which has historically focused on bacteria. Targeted modulation of the mycobiome holds significant promise for the development of innovative therapeutic approaches, ranging from antifungal agents and probiotics to FMT. However, these interventions require rigorous validation through clinical trials, and their mechanisms of action must be better understood. The identification of fungal biomarkers could revolutionize the diagnosis, monitoring, and treatment of neuropsychiatric disorders, bringing us closer to the era of precision psychiatry. Despite these advances, substantial gaps in our understanding persist. The complex interplay between fungal and bacterial communities, their interactions with host genetics, and their combined impact on neuropsychiatric outcomes remain largely unexplored. Addressing these challenges will require interdisciplinary research efforts that incorporate multi-omic approaches, longitudinal studies, and robust clinical trials. In conclusion, the gut mycobiome represents a new frontier in neuropsychiatric research, with the potential to unlock transformative insights into disease mechanisms and therapeutic strategies. By bridging existing knowledge gaps, future studies could establish the mycobiome as a cornerstone of personalized medicine in psychiatry, improving outcomes for millions of patients worldwide.
